# Beyond the 2-hour dogma: adsorption equilibrium versus saturation in polymyxin B hemoadsorption

**DOI:** 10.1186/s13054-026-06383-1

**Published:** 2026-10-08

**Authors:** Chizuru Yamashita, Yu Kato, Takahiro Kawaji, Mariko Nagata, Osamu Nishida, Tomoyuki Nakamura

**Affiliations:** 1 Department of Anesthesiology and Critical Care Medicine, Fujita Health University School of Medicine, Toyoake, Japan; 2 Oncology Innovation Center, Research Promotion Headquarters, Fujita Health University, Toyoake, Japan; 3 Tokyo Hikifune Hospital, 2-27-1 Higashimukojima, Sumida-ku, Tokyo, Japan

**To the Editor:**

We read with great interest the commentary by Legrand et al. [1] regarding the Tigris trial [2]. This trial was particularly important because its Bayesian design—drawing on prior evidence from EUPHRATES—provided strong statistical evidence for a mortality benefit of polymyxin B hemoadsorption (PMX; Toraymyxin™, Toray Industries, Tokyo, Japan) in a predefined population with endotoxin activity (EA) values of 0.60–0.89 [2]. As investigators involved in Tigris [2], Legrand and colleagues subsequently discussed the trial through 10 questions intended to help clarify its design and interpretation [1]. While we welcome this clarification, we believe that a mechanistic assumption embedded in their discussion of Questions 9 and 10 is not supported by experimental evidence: namely, that the endotoxin adsorption capacity of a single PMX-20R cartridge is limited to approximately 10–20 µg and that the cartridge approaches saturation during a single 2-hour session [1].

The 10–20 µg adsorption “capacity” and the concept of 2-hour “saturation” cited by Legrand et al. [1] are based on the review by Foster and Kellum [3], which in turn refers to experiments by Romaschin and colleagues [4]. A closer examination of these studies and the experimental data obtained during development of the PMX cartridge by Kodama et al. [5] reveals that the time-course experiments showing a plateau used a single 15-µg lipopolysaccharide (LPS) spiking in a closed, in vitro recirculating loop [4, 5]. In these closed-circuit experiments, as the LPS concentration in the fluid phase decreases, the system approaches an adsorption–desorption equilibrium. Consequently, the plateau in concentration reduction observed around 2 h is more reasonably interpreted as a dynamic adsorption equilibrium than as capacity saturation [4, 5]. In a separate experiment, Romaschin and colleagues spiked human whole blood with a much larger 100-µg LPS load and recovered approximately 20 µg of cartridge-bound LPS after 2 h; however, adsorption was assessed only at the 2-hour endpoint, so saturation was not established [4].

A PMX-20R cartridge contains approximately 300 mg of immobilized polymyxin B [5], and fully available 1:1 molar binding to LPS would correspond to a binding capacity more than 1000-fold greater than the 10–20 µg saturation limit assumed by Legrand and colleagues [1]. Although many factors may reduce binding, these simple stoichiometric considerations further challenge that assumed limit.

Our group previously addressed the issues debated here using a 24-hour continuous LPS-loading model with a neonatal PMX cartridge (PMX-01R; fiber mass 1/14 of the adult PMX-20R) [6]. Unlike the above-described single-dose experiments, LPS was continuously infused at 0.5 µg/h, resulting in concentrations in the sham circuit of 7.8 ng/mL at 2 h and 73.5 ng/mL at 24 h. Although adsorption efficiency declined after the initial 2 h—possibly because of protein surface adhesion—the endotoxin adsorption ratio remained approximately 40% thereafter, and cumulative adsorption increased almost linearly to 4.73 µg at 24 h without reaching saturation [6]. By fiber mass scaling, this would correspond to 66.2 µg of LPS adsorbed by a PMX-20R cartridge over the same period. Notably, the EA range of 0.60–0.89 studied in Tigris may roughly correspond to LPS concentrations of 2–4 ng/mL [3], well below those reached in our continuous-loading model. Our data therefore argue against premature saturation of PMX adsorption capacity within 2 h under clinically relevant endotoxin loads.

Most notably, prolonged PMX hemoperfusion is already practiced in Japan. By comparing plasma endotoxin concentrations before and after the clinical PMX-20R cartridge, Mitaka et al. (2014) demonstrated substantial endotoxin removal even after 24 h of treatment in patients with septic shock, with a median removal rate of 74.4% among patients with pre-cartridge endotoxin concentrations above the assay’s detection limit [7]. For acute exacerbation of idiopathic pulmonary fibrosis, PMX treatment for 6–24 h per cartridge is recommended in the approved Japanese labeling [8, 9], and this indication has been covered by national health insurance since November 2024 [10].

Given the importance of Tigris and its accompanying commentary for the future of PMX therapy, readers should be aware that the assumed 2-hour cartridge saturation lacks experimental support and is challenged by evidence to the contrary. The misconception may discourage consideration of prolonged treatment strategies and provide a misleading explanation for the apparent lack of benefit in patients with EA ≥ 0.90. In clinical sepsis, endotoxin is often released continuously, and prolonged perfusion with a single cartridge could offer a resource-efficient strategy for increasing its removal, potentially benefiting patients with particularly high endotoxin burdens. Although prolonged PMX treatment has already been investigated, its optimal duration and potential clinical benefits, particularly in patients with EA ≥ 0.90, remain to be established. It is time to move beyond the three-decade-old “2-hour dogma” [5] and reconsider how treatment duration might be optimized to improve endotoxin removal and patient outcomes.

## Data Availability

No datasets were generated or analysed during the current study.

## Abbreviations

PMX: Polymyxin B hemoadsorption
LPS: Lipopolysaccharide
EA: Endotoxin activity
